# Internet of Beer: A Review on Smart Technologies from Mash to Pint

**DOI:** 10.3390/foods9070950

**Published:** 2020-07-17

**Authors:** Simona Violino, Simone Figorilli, Corrado Costa, Federico Pallottino

**Affiliations:** Consiglio per la Ricerca in Agricoltura e L’analisi Dell’economia Agraria (CREA)-Centro di Ricerca Ingegneria e Trasformazioni Agroalimentari-Via della Pascolare 16, 00015 Monterotondo (Rome), Italy; simona.violino@crea.gov.it (S.V.); simone.figorilli@crea.gov.it (S.F.); corrado.costa@crea.gov.it (C.C.)

**Keywords:** IoT internet of things, microcontrollers, blockchain, craft beer, AI artificial intelligence, big data

## Abstract

The beer production chain includes some crucial steps regarding processing, delivery, service, and consumption that can benefit from the implementation of IoT (Internet of Things) based technologies. Large breweries implemented the use of sensors and digitization before smaller ones among which are craft breweries. Internet of Beer (IoB) technologies are becoming accessible to mid and small sized brewing companies. Therefore, the objective of this work is to review mainly low-cost IoB smart technologies that can be implemented from the mash to the final product and its service, to improve the brewing production, control, delivery, and final quality increasing profitability. The reviewed applications were retrieved both from the scientific databases and from the web. The work is structured in three macro areas such as beer processing, product logistics and traceability, and service. The results show a future trend characterized by a very fast increase in the use of IoB (also open source) systems to drive efficiency, productivity, quality, and safety. This will be done by real-time monitoring and a data-driven decision support system (DSS). Crucial aspects needing further investigation are data ownership and data standardization. The access price of IoB devices and software is destined for a significant decrease while their diversification on the market will grow leading to a massive future implementation within all the production levels.

## 1. Introduction

Beer represents a very important beverage being the first alcoholic drink consumed worldwide. Indeed, it is one of the oldest fermented alcoholic beverages in the world [1]. The origin of beer dates back to the 4th millennium a.C. in the East. Later it spread to Greece and ancient Rome, where it was not particularly appreciated. During Middle Ages it saw strong growth in northern and north-eastern Europe both in terms of production and diffusion, especially in monasteries. It had an exponential growth until 1914, while its production underwent a strong crisis during the First World War and in the following years. The 1930s brought a wave of relief and economic recovery. The advent of the Second World War, however, determined a new production decrease followed in 1950 by a period of recovery, as seen after the First World War. This recovery saw an increase in consumption and distribution especially in the 1960s and 1970s. Moreover, the 1980s represent a crucial period for the advent of modern distribution [2,3]. Bamforth [4] reports that in the last decades the technologies for malting and brewing significantly evolved by virtue of increased scientific knowledge. The product quality was improved and products differentiated, while the cost reduced in light of a lower environmental impact [5] and higher consumer safety. An interesting example regards energy consumption. A study carried out by Galitsky et al. [6] claims that energetic costs might account for up to the 38% of the total beer production costs. This is unlikely to be a representative number worldwide but gives an idea about the importance to improve energy efficiency in reducing the economic and environmental costs. The technical report suggests that given the available technology (considerably improved nowadays) there are still consistent opportunities to lower the energy consumption embracing correct brewing practices, monitoring the process, and implementing selected technologies. The same was done by Kubule et al. [7] who provided “a deep analysis of the potential impact of energy efficiency improvements by focusing on the evaluation of energy consumption and efficiency in various sub-departments at a small brewery”. However, it must be considered that the energy use, and relative carbon footprint, can undergo important variations according raw materials, size, processing methods, logistics, etc. Generally speaking technologies have already been implemented within almost all the crucial steps, including mashing, fermentation, even for real-time management to optimize cleaning operations in the brewery [8] or to evaluate the product quality through rapid test based on advanced computer vision and image analysis techniques [9,10].

However, these technologies are often a prerogative of large and medium-sized manufacturers due to the knowledge needed for their management, development, and implementation costs requiring specific planning. As a result, the rapidly growing artisan sector often does not have adequate employees and funds to adequately implement these technologies.

Currently, the world beer market is rapidly evolving especially due to the raise of the craft beer sector innovating in terms of product differentiation and quality. As a matter of fact, independent craft breweries put emphasis on new flavors and product retrieving old beer styles and ancient production techniques which strengthen the sector itself creating interest for the consumers. In some cases it is possible to talk even of commercial nanobreweries [11]. This trend, known as “Craft beer revolution”, was registered in the US first, since 1980, and afterwards in many other European countries such as Germany Italy, Spain, UK, etc. [12,13]. The trend shows as consumers started looking for quality products; since then their preferences regarding beer industries changed. The industrial breweries produce large quantities of beer, while craft breweries produce small quantities, and therefore can easily produce different types of beer, changing malt, hops, yeast strains, etc. Craft beer is usually not filtrated or pasteurized with more characterized taste and aromas. These represent some of the reasons why always more consumers are attracted to these beers and consider them of higher quality with respect to industrial ones [14]. However, quality needs to be addressed by pursuing an adequate technological level that can be crucial in maintaining high standards over time.

Nowadays on the market an increasing number of open source systems (software and hardware), digital smart devices, and applications (also mobile based) are available. These are normally designed, or predisposed, to interact with each other as an Internet of Things (IoT) system. These can often be implemented aftermarket at relatively low prices within many production systems, including the beer sector, to accomplish several tasks [15,16]. Many of these technologies, specifically built for brewing, have been firstly developed and applied at homebrewing or pilot level so they will need to be rescaled to be suitable even for a small craft commercial brewery. Nevertheless, many of such systems benefit from a community that supports the development and the adaptation to real-case applications. Although the companies building beer production systems offer already in many cases technological solutions, these are often closed systems that need dedicated assistance and suffer from limited interface with latest technological systems. Moreover, these often come at high costs.

Thus, this work aims to review low-cost IoB (Internet of Beer) smart technologies that can be implemented from the mash to the final product and its service, to improve the brewing processes, control, delivery, and final quality. It must be said that several of these systems are unsuitable for handling certain tasks and some of them require a certain degree of knowledge. Internet of Beer (IoB) definition has been recently introduced and the authors find it more appropriate with regard to Internet of Brewing since it includes aspects related to the whole beer chain (e.g*.,* logistics and service) and not just those related to the product manufacturing. The present work mainly tries to uncover low-cost technologies that might be applied to mid-sized and small breweries, normally representing the craft sector, but embrace some higher cost applications due to their impact and innovativeness.

## 2. Bibliographic Retrieval and Brewing Main Process

**Figure 1:**
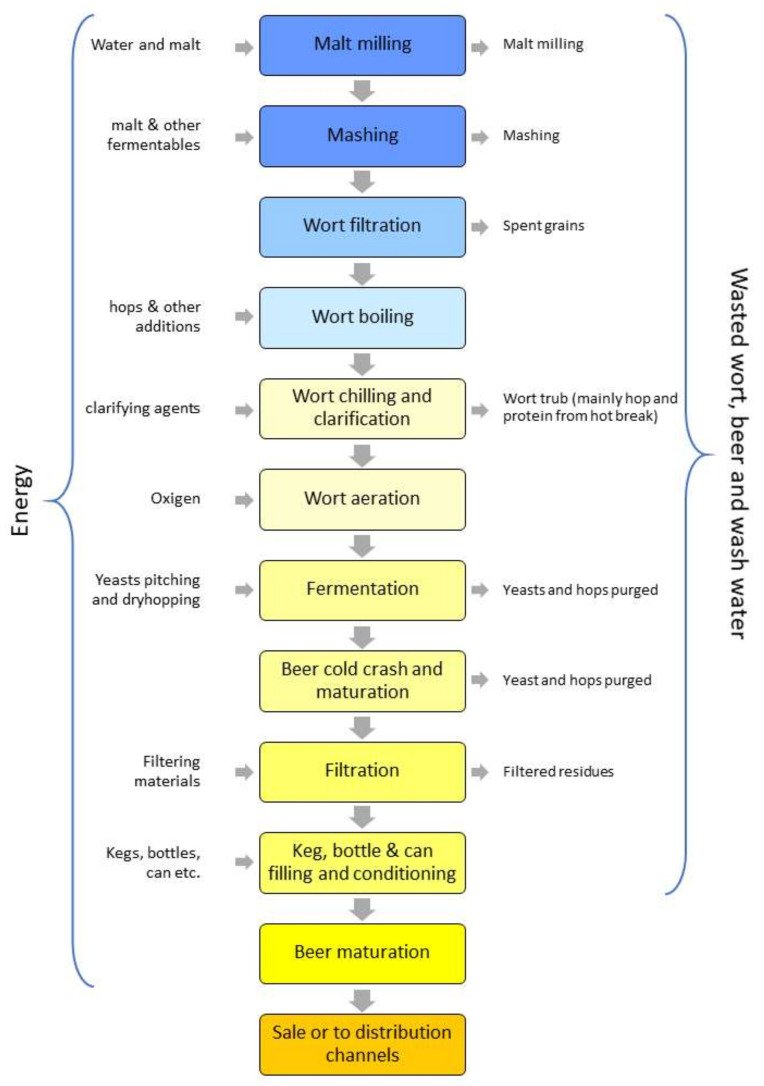
Brewing process and relative input/output involved inspired by Nimbalkar et al. [16].

The work represents a review of the emerging low-cost smart technologies used in the beer chain from brewery management operation to the product draft service. In detail, the bibliography describing the applications presented in the work were retrieved from both scientific databases and directly from the web. Scientific literature regarding beer and these kinds of applications are not exhaustive and therefore insufficient alone to review these technologies. Example of keywords used to perform the research through Google Scholar, Scopus, and main Google search engine are IoT and beer, IoT and brewing, IoT and a series of terms relative to specific production phases of the brewing process (e.g., mashing, filtering, etc.). Moreover, were used terms related to the whole beer chain paired to specific devices normally part of the applications (e.g., Arduino, Raspberry Pi, sensors, etc.). The research was undertaken with regard to three main fields which are:

1. the whole operations involved in the brewery including inventory management, finished products in stock, the real production process (Figure 1), and the Cleaning In Place (CIP);
2. traceability and logistics;
3. product service.

## 3. IoT and Relative Technologies

Today Internet of Things (IoT) is a widespread definition indicating a set of “smart objects” (smart devices) connected to and through the internet. Through the connection, information is observed, recorded, analyzed, and transferred, in a context in which “things” talk to each other and then carry out consequent actions. The origin of the term is attributed to Kevin Ashton, in 1999, in a presentation made for Procter & Gamble. Kevin Ashton, raised how the initial idea of the term was still not well understood, focusing on in what way almost all the information is generated by humans and specifying the enormous potential to have “objects” able to detect, through sensors, the surrounding world, for us [17].

Nowadays we have achieved most of what he pointed out. In this new era, we have a set of electronic devices, often man-independent, equipped with various sensors capable of detecting the surrounding world and sharing what has been observed to make decisions.

This evolution, if not technological revolution, is made possible by the enormous progress in the context of consumer electronics and wireless communication. The advent of the Open Hardware concept, the availability of increasingly powerful and less expensive microcontrollers, have started a process of maximum sensors capillarity and embedded technologies, enhancing in fact the “things” connected to the Internet. An IoT device needs three fundamental elements divided into macro-areas concerning (i) the microcontroller or processing unit, (ii) the sensors and/or the execution capability, and (iii) technology for the connection to Internet. The elements belonging to these macro-areas can be found, to date, in most devices, not only within specific application context, but also for domestic applications such as in a camera, a thermostat, a vehicle, etc., for the process of remote management and control.

The most popular boards used by IoT applications tend to directly integrate a radio module. Arduino MKR WAN 1310 for example is equipped with LoraWan technology while Raspberry Pi 4 with Wi-Fi technology and Bluetooth with possibility to interface sensors and actuators. The most widely used sensors find their place in an environmental monitoring context, thus, there are a multitude of temperature and humidity sensors on the market.

In the agrifood sector, many environmental monitoring cards are equipped with sensors to measure temperature, humidity, soil moisture, rain, UV, etc., with the addition of actuators for the control of solenoid valves and/or switches. Industrial context uses IoT devices for transferring the process to the network, expanding the potential for remote management and control while reducing man work.

IoT technologies and devices are applied to a wide number of sectors with a steep growth [18]. Their enormous diffusion has given rise to dedicated networks that differ from classical ones for the amount of data to be transferred, the distances to be covered, or limitations of available energy (battery devices).

**Figure 2:**
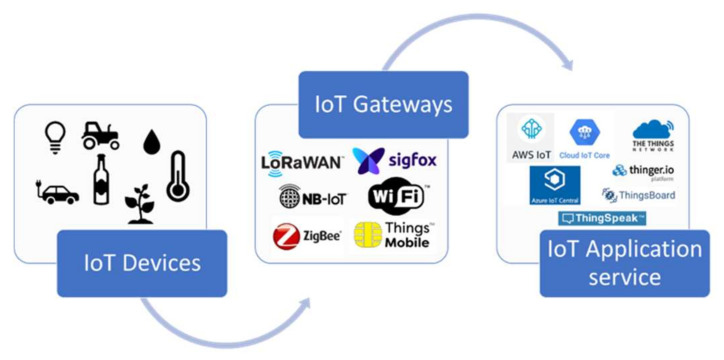
Scheme of the levels of the Internet of Things (IoT) infrastructure.

Moreover, to interconnect IoT devices specific infrastructures (e.g., cloud services) have been designed and developed according to different visions and needs. These infrastructures can be divided into three levels normally common to all the existing implemented forms (Figure 2). The first level includes the devices that collect the information and interact with each other and/or execute commands; the second level is represented by the IoT Gateways, whose fundamental role is to be the collector of all the devices, the various network typologies reside here (e.g., Lora, sigfox, NB-IoT, 3G/4G/5G, WiFi, and ZigBee); finally, the third level is represented by the IoT application service implementing all the logistics behind the management of the information coming from the devices. This regards the whole set of services including DB (Database), application, and dashboard for Man-Machine interaction. The third level includes all IoT platforms, from the most complete ones, offered by cloud services, to open-source web platforms [19].

The increasingly popular IoT platforms today offer the same basic services aimed at creating dashboards, managing and storing and exporting data, managing alerts and notifications, up to a higher level offering statistics and data analysis, projecting the systems close to big data techniques available nowadays. By communicating with the IoT Gateways level they can receive data from the devices and provide DB services. Nevertheless, through specific graphic elements, they provide many tools to simplify the real-time interaction and consultation of the system state and information. The whole system is commonly accessible remotely, in real-time, and allows historical data queries.

Among the best known IoT platforms there are the Microsoft IoT Central based on Azure cloud, the Amazon Web Services (AWS) IoT of Amazon cloud, and Google’s Cloud IoT Core. These are the proposals offered by the modern big names in information technology; thanks to the potential of their clouds, they make available a set of services targeted for the IoT, that can be easily integrated with the whole cloud world. In addition to proprietary platforms there are open source solutions. These, expanding their services with connectors, mainly offer targeted services for the IoT, from device management, data logging, and the control dashboard, to specifically targeted managements towards the IoT Gateways level. Table 1 reports a comparative scheme of the best IoT open platforms [20]. The parameters taken into consideration concern the main characteristics of each platform such as the device management, security, the communication protocols towards the devices and other software environments, the ability of the platform to perform data analysis, and the integrated database service.

In the context of IoT, the open source concept plays a fundamental role. The term that was firstly introduced in 1998, when Netscape decided to publish the source code of its Navigator browser [21]. Nowadays, this concept has undergone a division, creating two macro-categories, identified as open source software and hardware. The IoT field embraces an entire hardware and software ecosystem, including devices, network infrastructures, and software platforms. Therefore, it is important to address the world of open source, to distinguish and consider the open source hardware and software separately. The first category regards the devices, the considerable growth in consumer electronics, is mainly attributable to the release of devices under open source hardware license. The second mainly aims at the platforms, that being “open”, allow to create a customized IoT infrastructure, molding it to specific needs. Although the open source hardware and software are very different in terms of composition, they have common aspects related to the advantages and disadvantages that can be broadly summarized by the following aspects.

Possible benefits that could derive from their use regard: (i) no/reduced cost, (ii) large community of developers and/or supporters, (iii) accessible source code (that can be modified and commercially used for profit). The disadvantages instead may regard: (i) regularity of updates at risk if the product is no longer supported by the community, (ii) ancillary costs to ensure correct operation, customizations, and modifications of the source code, (iii) often higher technical skills required.

Finally, thanks to the open source philosophy, it was possible to observe the evolution of the IoT world, leading to the emergence of diversified devices and platforms, for every working context and not, making the world increasingly connected [22].

At last, regarding the security aspect concerning the data involved with IoT systems, a series of technologies are gaining always more attention being naturally suitable for the scope. These technologies include blockchain [23,24], RFID (Radio Frequency Identification), and other used for traceability purposes [25,26].

## 4. Applications Retrieved and IoB Technologies

The ultimate and ideal scope of these technologies is to attempt to connect the entire brewing process using digitally linked hardware and software from the beer design and conceptualization to sales report following a feedback loop scheme.

It is necessary to say that at the moment, the benefits coming from the use of such technologies are mainly retrievable from producers claims and individual use cases but lack of scientific support and literature quantifying their advantages. However, in light of the strong and increasing interests, sales, and the digital implementation within other sectors, these represent valuable reasons to consider the reported applications.

### 4.1. IoB Technologies for the Brewery

To improve and constantly produce high quality beer, all the breweries, including craft ones, are starting to use IoT sensors or digital implementations. The IoT devices are becoming part of our daily life. Internet of Things devices allow the collection of crucial and valuable information that helps in a variety of fields: from analyzing the evolution of crops on agricultural land (barley grown for malt production) to monitoring how users consume a certain product and if they appreciated it. The application of IoB (Internet of Beer) technology can begin even with the production of cereals thanks to the installation of soil sensors that allow to monitor crops and predict the treatments to be carried out [27]. During the production phase it is important to monitor several factors influencing the processes. For craft breweries, some stages of the production chain could present higher problems with respect to industrial breweries. These for example include the impossibility to monitor the fermentation process during the night and during the weekends, this commonly being manually controlled. In this phase, the yeast consumes the sugars, increases the alcohol content, and decreases the liquid density. Besides these aspects, temperature variations outside the prefixed range can negatively affect the final aroma stimulating an excessive production of esters or other aromatic compounds. To prevent these problems, wired sensors are used in industrial breweries. In craft brewing, due to often not optimized installations, limited space available, and budget problems, wired installation can be problematic. Furthermore, cabled systems have defects related to the contamination and size of the equipment used. An innovative practice is represented by the use of Bluetooth sensors that now provide long-range functionality and easier installation. To overcome indoor flow problems, Cassia Networks used long-range Bluetooth sensors (battery powered) boxed inside acrylic tubes easy to sterilize. These can be inserted inside the steel unitanks, floating in the wort. A long-range Bluetooth router connects to the low energy Bluetooth sensor monitoring density and temperature of the liquid inside the tank. This method, in addition of being certainly more pragmatic than wired sensors, is low-cost and therefore accessible to microbreweries, where operators through smartphones or tablets can monitor production parameters remotely. Currently, the tablets and smartphones are all Bluetooth Low Energy enabled and represent a COTS (Consumer Off-The-Shelf) method compared to other expensive monitoring equipment [28].

Normally industrial big beer groups use proprietary software while average to small breweries rely on commercial software or even free/open source ones used at homebrewing level. Some of the software available nowadays aggregate a lot of information and represent the communication structure for sensors and other devices. Please note that some of the software reported below include also tools very helpful for the traceability and logistics but being in the first place used for brewing and batch management they are reported in the present paragraph.

An example is represented by Orchestratedbeer [29], a complete solution including modules dedicated to the management of activity planning, inventory, production, purchases and sales, accounting, quality control, reports, and dashboard for each step, all in real-time. While the information used by the software are acquired through sensors (e.g., fermentation temperature) some others need to be manually inserted by operators. This aspect is common across many software and some of them completely rely on manual data entry. BeerifyMe! [30] is another valid software which works similarly including also customer’s management and cloud solution 24/7. The platforms available for both are numerous (pc and mobile based). Ekos brewmaster, like its siblings, automatically calculates the production costs, while continuously recording data relative to the brew, fermentation, and conditioning logs for beer production and relative equipment maintenance. Brewplanner [31] instead focuses on tasks coordination and the specific effect of an action on other areas in order to understand the limitations and capabilities of the brewery and to apply real-time correction. Iconic^®^ BMS [32] offers a set of features for a 360° management software that include also mobile POS tools enabling to handle B2B sales directly on the road and B2C sales at events or in the tasting room. VicinityBrew [33] claims to be specialized, in comparison with other software in multi-stage production runs since, in the producer’s opinion, most systems are not able to do that. The software can manage interdependent production runs and generates specific report outputs for each stage. Interestingly, it offers dedicated tools for “contract brewing” helpful to manage batches done for beer firms.

Besides these comprehensive commercial softwares, of which just a small number was cited, there is a huge number of smaller software mainly aimed at small breweries and homebrewing applications. Generally, the main goal of these tools is to provide the environment for managing inventory, planning recipes, following and recording the batches until finished products, associating information related to the beer styles defined by the Beer Judges Certification Program (BJCP), [34], importing/exporting recipes files, and writing notes. Valid examples are Brewtarget [35] completely free and open source, BeerSmith 3 [36], Brewfather [37] and many more upcoming or discontinued like e.g., the well-known in the past ProMash [38] or open source HobbyBrew [39]. Among all, Brewfather is characterized by a modern look being structured as a webapp or mobile App. It is probably the only software, at this level, which at the moment offers a solid integration with a growing number of wireless or Bluetooth devices mainly suitable at pilot and homebrewing levels.

**Figure 3:**
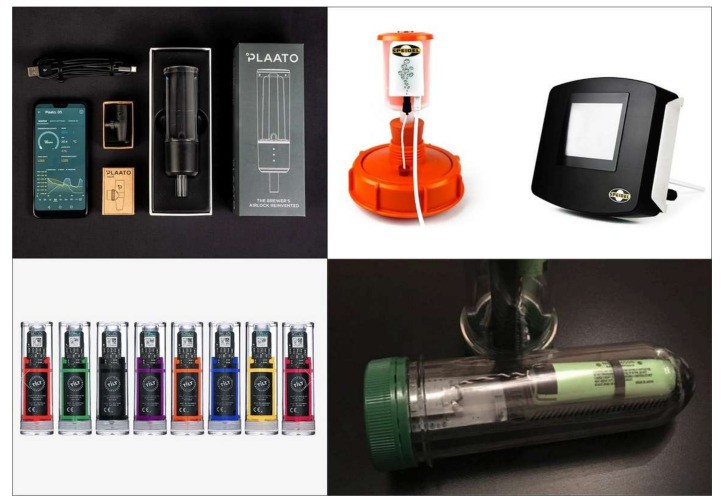
Devices. **Top left**—Plaato Airlock (image source: https://plaato.io/products/plaato-airlock)); **Top right**—Speidel GÄRSPUNDmobil (image source: https://www.speidels-braumeister.de/en/braumeister/gaerspundmobil-and-gaermeister-control.html); **Bottom left**—Tilt Hydrometer (image source: https://tilthydrometer.com/); **Bottom right**—iSpindel DIY electronic Hydrometer (http://www.ispindel.de/).

The supported devices include Plaato Airlock [40,41], a digital airlock that can be easily mounted on the fermenter in place of a normal bubbler to continuously measure the CO_2_ flow (counting every single bubble) and converting it to information inherent specific gravity, ongoing fermentation, alcohol percentage, and the ambient temperature (Figure 3). It includes three LEDs indicating the CO_2_ pressure. The information is sent from the Plaato device to a smartphone App via Wi-Fi for real-time monitoring and stored for later use and batch comparison. The same principle is used by the GÄRSPUND mobil from Speidel [42] allowing you to monitor the fermenting process remotely (Figure 3). As in the previous case, the device counts the number of bubbles from the fermenter, monitors the temperature through a specific temperature probe, and sends data to a MySpeidel account over Wi-Fi. Speidel claims that the data collected by the GÄRSPUND mobil, that can be paired with the Gärmeister fermentation control device, can enables to supervise and analyze the fermentation to predict and plan the bottling date, and to correlate the fermentation and the temperature curves with the taste of the final beer optimizing the brewing process. Similar tasks are accomplished by the open source device iSpindel [43] and Tilt™ hydrometer [44] which however work through a different principle. Both the devices work through a Bluetooth connection and include low-cost components. The systems are built around the concept of a tilting cylinder without the need of any external reference except gravity (Figure 3). The cylinders are easy to keep clean and to maintain. Their inclination angle changes in relation to the buoyancy and thus is directly related to the liquid density and therefore the sugar content. Calibration can be applied whenever necessary or when batteries are changed. It is interesting to consider that these last devices can be used in fermenters of any size (if there is enough signal through the insulation walls) while the first must be the size of the base of the fermenter. Devices such as the cited hydrometers and thermometers monitor the fermentation relying on a smartphone, but a valid alternative is represented by single board pc like the Raspberry Pi [45] or Arduino [46]. These are small single-board computers used all over the world for open source (and not only) applications for robotics, research projects, etc. Their success is due to extreme portability, very high flexibility, and extremely low cost. Thus, sensors can be read setting up a webserver on one of these or through specifically developed devices e.g., MyBrewbot [47] representing a complete fermentation telemetry solution for brewing. Another interesting device, which is implemented within Brewfather, and very rich of features, is represented by SmartPid [48]. This controller has been designed, with several dedicated applications among which one is for brewing. In the specific case the Smart homebrewing app, once installed, lets you manage the brew automation for BIAB (Brew In A Bag), RIMS (Recirculating Infusion Mash System) and HERMS (Heat Exchange Recirculating Mash Systems) both, manually or in full automatic mode. Moreover, it supports multi-channel management for mashing and hot liquor tank, fully configurable PID-PWM control and ON/OFF control with hysteresis, heating or cooling, IoT connection via WiFi to cloud services and remote control via Message Queue Telemetry Transport (MQTT) standard protocol, full compatibility with the Arduino ecosystem. A similar one, but with different features is the AxHTherm smart controller [49] specifically designed for fermentation chambers that can be can remotely managed with the aid of BrewFather and/or ThingSpeak integration.

Another tool that allows to control mashing (including pumps) and the beer fermentation temperature with an accuracy of 0.1 °C is BrewPi Spark 3. The BrewPi Spark 3 has the ability to read, through sensors, the temperature of beer and a refrigerator working as fermentation chamber switching on and off the heater and radiator when necessary. In the Spark, all temperature control algorithms work locally; in this way it is completely autonomous to control the beer processing but integrates the possibility to monitor the process from a smartphone or tablet. A Raspberry Pi board can communicate with BrewPi Spark over USB connection for recording the delivery data. Moreover, it has also a web interface to monitor and control the process via the browser. On the BrewPi web page, you can start and stop a mixture, change the temperature profile, view graphs, and change settings [50]. The system is powered by the open source software project CraftBeerPi, based on Raspberry Pi hardware, supporting a huge number of customizable applications from mash to fermenting with preset or custom profiles [51,52]. Raspberry Pi, Python language, and Linux OS are well known open source solutions often used for these scopes [53].

All the above-mentioned devices can use several communication strategies. Zhao et al. [54] experimented and tested a fermentation control through an IoT system based on wireless sensors communicating though a ZigBee network. The authors firstly analyzed the IoT technology available and the hysteresis process of beer fermentation, and then designed the beer fermentation control program. To do so, the chip CC2430 was used to achieve automatic detection and control of beer fermentation. The research showed the wireless network control structure of ZigBee can be simple to set up offering flexible communication at low costs. It concludes underlining the potential of the tested system in reducing the investment needed for the beer fermentation and human capital to complete the tasks. In fact, there are already examples of feasible applications relying on Wireless Sensor Networks (WSN). This is testified, for example by the experience of Queen City Brewery [55] that implemented a real-time, cloud based, fermentation monitoring solution using the LORD wireless sensors [56].

However, wireless sensors are not exclusively used by craft breweries. Normally, fermentation is represented by a curve whose shape is determined by the recipe and can vary depending on the yeast activity which is not always predictable. Yeast can be more active in some batches than others and can degrade over time if more generations of the same yeast are used. In the case of the MillerCoors company, the fermentation data showed that it has ceased, and the density stopped decreasing long before the original fermentation program, often 40–50 h in advance. Through sound density sensors (patent pending) combined with TZEROBrew software, is possible to monitor the fermentation status in real-time. Hence, TZERO offers a digital version of the fermenters so that in few seconds it is possible to visually understand the status of the entire fermentation process [57]. Precision Fermentation company claims to offer the first world real-time monitoring complete solution to enhance control over the fermentation process. Their flagship product, the BrewMonitor^®^ System, is advertised to potentially raise business profitability by monitoring pH, density, pressure, conductivity, dissolved oxygen, and internal/external temperature [58]. Pentair [59,60] launched a new Internet of Things (IoT) solution for beer membrane filtration (BMF) systems. This, following the producer claims, enables brewers to monitor the filtration process performance, optimizing efficiency and improving the final product quality while lowering operation and upstream costs.

Furthermore, another technological advancement in IoB is represented by all in one IoT brewing systems suitable for (mainly) homebrewing and real commercial production. There are several of them available on the market. Brewbot is a high tech small-scale brewing device IoT enabled. As reported by Samuel Khamis (chief science officer at Brewbot), beer production can take about 4.5 h. The Brewbot is able to perform a series of steps independently, communicating with the user via the Brewbot app. The device targets large-scale breweries, for pilot plant purposes, to test new recipes. The system reaches a maximum production of 45 L [61]. Other systems, that work similarly, include the Grainfather [62] producing up to 70 L, Brewtools up to 150 L [63], Speidel Braumeister which has a maximum of 1000 L [64], and many others. Their aims differ on the basis of the production potential, from 150 to 200 L a nano brewing system can be potentially useful for a restaurant or a small brewpub, from 4 to 500 L of finished products onwards can work for a microbrewery.

An interesting and very new trend is the creation of micromalting systems equipped with sensors, real-time data reading, display, and remote control. As a matter of fact, precise control is fundamental for proper malting from kernels germination to roasting. Sprowtlab [65] created the Acro remote controllable small malting system relying on a dedicated software to track and use data in order to produce high-quality malt. The same has been done by BBC Inox [66] that produces micro malting systems starting from higher production level with a minimum of 100 kg of finished product per batch till 4000 kg. A smaller, promising system is being developed by Arzaman S.r.l. [67] starting with a system able to produce around 10–25 kg of malt including special ones due to the implementation of a drum roasting element. The system relies on the SmartPid controller described above presenting very high process control precision. It is Wi-Fi connected, so even when the operator is not closed to the machine there is the possibility to monitor the process through web app to read real-time malting data.

Another interesting aspect regards the objective quality control these technologies can enable which is often a prerogative of big breweries. Di Caro et al. [68] proposed a low-cost spectrophotometer for the measurement of beer color in relation with the European Brewery Convention (EBC) international method. The color is a crucial quality parameter for the product evaluation, it is usually measured by means of spectrophotometric techniques that have been adopted as international standard by many brewers’ associations in the world. The study proposes a low-cost instrument that could be used by many breweries that cannot afford expensive devices.

Finally, Nimbalkar et al. [16] studied the use IoT technologies for the breweries and underlined that these are crucial to obtain relevant real-time, and for later comparison, information inherent every stage of the production and sales processes. The authors agree on how modern breweries can use a computing and communication core system based on IoT to monitor, coordinate, control, and integrate physical and engineered systems. Moreover, they pointed out that the interactions between humans and systems create dynamic networks potentially improving cost structures and resource utilization.

### 4.2. IoB for the Traceability and Logistic

Traceability is usually defined as the ability to follow the movement of a food, through its entire supply chain, from production to distribution and product location. Currently, many food companies rely on IoT systems such as smart tags (RFID, NFC, and barcodes) to obtain traceability information [25,26]. In the beer sector, transport is a phase of the supply chain that needs to be monitored. It is crucial that the product arrives as quickly as possible from its place of origin to the table and above all in optimal conditions, preserving its original qualitative characteristics that can deteriorate when the product is exposed to excessive temperature and direct sunlight. This is particularly true for craft beers that are generally not pasteurized or heavily filtered e.g., with diatomaceous earth (fossilized marine alga diatoms).

The transport phase along the beer supply chain is not only important for the finished beer but starts with hops extremely vulnerable to temperature and oxygen. This freshly harvested primary ingredient has a short shelf life, so it must be transported to the production plant within a short time. To overcome eventual problems, Rogue Farms (Newport, Oregon), through the installation of intelligent sensors, is able to monitor and trace the hops, controlling its temperature and humidity during transport and estimating the time of arrival. The shipment of hops is controlled through a “visibility platform” (Intel), which allows to receive an alert on the status of location every 10 min and when the product is halfway through, the plant begins with the production phase. In this way, the manufacturer is able to target the processing time to make the hops use coincide with its delivery and thus to reduce the deterioration of the raw material [69]. IoT technology are not only important for the beer traceability during the production process, but also when the barrels or pallets of beer are ready to be shipped. In this regard, the US beer distributor, B United International, uses satellite sensors (Ovinto) to monitor the position, temperature, and pressure of hundreds of brands of beer, cider, and mead as they are shipped to breweries around the world. Thanks to sensors relying on satellites for positioning it is possible to update in real time the state of the beer of each container, even if in transit across the ocean. As a result, real-time data will be shared with business customers and consumers. The IoB system minimizes beer exposure to pressure and temperature fluctuations, with the result that complex naturally refermented beers will taste in the end point as similar as possible as they did when they left the brewery in their home countries [70]. In recent years, the new “Bock Chain” beer has been brewed. The beer relies on a blockchain system to track ingredients and beer from field to can. The can has a final QR code on the label, which users can scan with their phone to retrieve details about the product and its origins. The QR code unlocks a microsite containing videos, photos, maps, data, timestamps related to the entire beer chain. There is a time-lapse video showing the barley growing in the field and animated maps illustrating various stages of the supply chain. Each step contains photos, videos, and descriptions of the processing, including analysis. The microsite also allows consumers to share their Bock Chain experience through a social link [71]. In addition, to reduce fraud, theft, and losses related to barrel and keg tracking, a platform has been created to automate the keg tracking process, i.e., Asset Tracking Platform based on Blockchain and IoT. The platform uniquely identifies each keg/bottle and its content and captures important data throughout the whole supply chain. Using the Blockchain enabled system, breweries get accurate and up-to-date information and reports about keg filling, status, movement, customer location, returns, needs for keg repair and maintenance. The structure was created on a private blockchain based on Hyperledger, so there is no need for intermediaries. The solution allows customers to create intelligent contracts to improve transparency in their supply chain by recording the origin of barrels or casks [72]. Another use case of Blockchain paired with IoT is represented by the system developed by oracle and embraced for example by Alpha Acid Brewing (Belmont, California, CA, USA). Oracle claims their Blockchain Applications can enhance traceability and transparency throughout the value chain. The application includes a set of services enabling end-to-end traceability of goods and transactions in the supply chains, product genealogy and provenance, monitoring and tracking the temperature along the whole supply chain, creating recommendations to optimize processes, and much more [73]. Kyle Bozicevic, Alpha Acid Brewing Company owner said “With Oracle Blockchain solutions, we can track materials and ingredients from our suppliers and analyze sensor data from the production process. Oracle Blockchain Platform tracks where we are getting the highest quality hops, malt, and yeast, and enables us to create a strong narrative around our products” [74] so the system help as well in creating a storytelling to enhance the marketed products. In addition, the consumer can read all the information with a smartphone scanning a QR-code and give a feedback helpful to identify and solve problems where and when he is not satisfied with the product [75].

In general, breweries are relying on IoT and blockchain technologies to improve profits, optimize product quality and transparency towards consumers. IoT enables them to control technical problems and malfunctions along the beer supply chain. Through a system equipped with sensors, conditions can be monitored and notified in real time during delivery, reducing waste and costs for companies. At the same time, the blockchain applied to food traceability allows all participants to see the data recorded in the previous steps, which means that customers will be able to track their food. The system collects and provides customers, producers, and anyone involved in the network with detailed information on a product and its traceability. The end user will be able to access it by scanning QR product codes and other types of labels [76].

Hershberger et al. [77] patented a system (based on RFID and pressure sensors) for the brewing industry logistic to improve the low efficiency of the traditional barcode to speed up the product identification and kegs filling level. This is just one out of many technologies that need to be combined together to create a digitalized supply chain. A study conducted by VLB Berlin [78] on the “Digitalization and trends in beer logistics” concludes that this digitalization needs some internal and external changes to be effective. Among the first cited is the necessity to equip the warehouse with technology to achieve a digitalization of the material/products flow, to automate the identification of products for precise monitoring of the batches, to apply RFID as a full identification solution, and to implement smart devices (smartphones, smartwatches, and tablets). Among the external changes that need to be made, the study cites the importance of a cooperative approach and data exchange between manufacturers and wholesalers through digitalized and standardized processes.

The cooperation among stakeholders, and thus the information standardization, is also one of cores of big data and AI applications depending on data acquired through IoT systems to improve sales and the efficiency within the supply chain. Heineken uses big data and AI on data regarding various beer brands it owns to increase efficiency and manage its operations, marketing, advertising, and customer experience [79]. Anheuser-Busch InBev, at the moment the world’s biggest beer company, is undergoing a huge digital transformation project trying to amalgamate data from dozens of independent breweries of their properties into a unified data central structure called Enterprise Data Hub (EDH) using data to enhance their business processes, improve consumer relations, and even to make better beer [80]. The platform, running in Microsoft Azure, consists of a blend of Microsoft and third-party utilities, uses several layers of data, including layers for raw, clean, and optimized data. The infrastructure supports different use cases including demand forecasting, fraud detection, social media listening, and IoT analytics. In an interview to datanami [80] the group declare that the data generated by brewery equipment basically have the same indicators, so they are easily scalable to all breweries.

### 4.3. IoB for Beer Service, Marketing, and Consumption

Probably, the least exploited stage through the IoT, and therefore one of the most innovative, regards consumers’ behavior and feedback. Knowing when, how, and how much your product is consumed and liked is valuable information and a very powerful tool for any company. Geeksme (a Spanish company) for example, is working on an IoT device that, among other things, will allow brewers to “sensorize” beer taps and bottle openers so they can extract information about their customers’ consumption [27]. There are different types of IoB technologies that are starting to spread around the world in this direction. Taptronics from Pubinno, is a technological system that allows the bartender to be informed about the content left in the keg as a clever, connected plug and play beer tap. This technology tries to serve ideal beer, monitoring temperature and pressure in one touch in every single glass. In addition, the producer claims how its use may save up to 20% of the costs to restaurants and bars reducing waste and fraud. Pubinno was also the creator of BeerPoint technology, an IoT-enabled self-service beer vending machine that uses prepaid NFC cards sold in stores. Customers, by purchasing NFC cards, can obtain their drink with a simple tap. Serving a glass of beer in less than a minute reduces waiting time and increases customer satisfaction [81,82]. An innovative IoT sensor was also designed by Robiotic. This technology is basically a coaster that uses a strain gauge membrane sensor to determine the level of beer (evaluating its weight) in a glass placed above it. When the coaster determines that the customer have drunk enough liquid and need a refill, reports the information directly to the bartender or using a server, wirelessly. Given an RFID-enabled beer glass decorated with your favorite beer brand, the coaster can even signal the beer brand you need to refill [83]. The beverage manufacturer Carling has also launched the world’s first “Beer Button”. It is a technology that allows you to refill an empty fridge by connecting drinkers to online shopping baskets from the comfort of their own homes, exactly what Amazon did with its product specific “Dash Button”. The Beer Button connects to a smartphone app, which in turn links to the user’s account at a retailer of their choice. Customers only need to press the button to automatically add beer to their shopping basket and buy [84]. Another IoT product that allows you to optimize your beer service is the Kegtron. This is a tool that allows to precisely trace the levels of the drums, displaying the information on your mobile device. It consists of a small box that is placed in line between a barrel and the tap to which the barrel is connected. The box hosts a flow meter that accurately measures the amount of liquid dispensed a processor and a wireless module that sends immediate updates to any device on which the Kegtron application is installed (available for both iPhone and Android devices) [85]. Another (patent-pending) machine-to-machine communication system for accurate inventory and beer order processing is SteadyServ’s iKeg. Through the iKeg application, retailers can also know exactly what they have in their cooler and which draught beer is the most consumed to ensure that the taps are turned efficiently. A correct tap list needs to observe principles regarding proper beer differentiation. Orders can be placed via voice, text, and e-mail through the iKeg application. Retailers can find out when the beers are running out and when they need to re-order. The iKeg app also allows you to send automatic, customizable social alerts on Facebook and Twitter whenever a new draft beer is available. It also provides a fully integrated set of tools to advertise a club, a special event, or a unique beer. A similar technology is represented by iPourIt. Customers, once they enter a bar or pub, leaving their credit card details, receive a wristband with RFID technology. As he pours a beer, the sensor in the handle of each keg that reads the ID of the unique bracelet, and a valve connected to the tap that measures the amount of beer he pours, allowing the company to charge per ounce poured [86,87,88]. An innovative device not yet on the market is the WECHEER.IO, a gadget for beer lovers. It is an intelligent bottle opener, able to instantly identify the person, time, place, and brand of any bottle with crown cap. Working via Bluetooth, WECHEER.IO allows users to share their favorite moments and helps them keep track of their movements the night before. It is speculated that it may cost $55. Surely it is a pretty expensive item, designed more for restaurants than for individual customers [89]. The researchers were from the Technical Research Centre VTT and the Finnish company UpCode, within the European project TagItSmart!—funded by the Horizon 2020 programme. They collaborated to introduce reversible functional thermochromic sensors and inks in consumer goods, such as beer. These thermochromic inks reacting to light and temperature variations, are made of photonic liquid crystals and leuco dyes. Placing these intelligent sensors on beer labels, the consumer knows whether the beer can be consumed and the circumstances under which it was brewed. The smart beers are currently on sale in a neighborhood shop (Vaasa, Finland) to track the opinions of Finnish consumers [90].

Another interesting solution is represented by “Simple Beer Service” application and hardware. The idea developed by Amazon Web Services Startup Program [91] is to use a Raspberry Pi together with a kegerator (draft beer system) to measure when a pour is occurring, post the data to Amazon API Gateway and on a dedicated web page through a dashboard written in Java. This open source system [92] can be applied and scaled up for use in a pub. It demonstrates how informative and affordable these kinds of technology and services can be [93]. An alternative open source system showing similar features is represented by Kegbot [94]. The system enables real-time keg monitoring to always know how much beer is left in the kegs tracking in detail the history of what is poured and building a management database. Moreover, being open source results to be very extendable and well supported by the developer community supporting the project. The system final price is reasonable and needs just a sensor board, a flow meter, the necessary barbed fittings, an RJ45 ethernet, and a micro USB cable. To operate the system requires an android smartphone, or tablet for better viewing, with the installed dedicated free app showing keg status, statistics, and pictures taken (e.g., indicating brewery logo and/or beer label). A more advanced system is I-TapR2 [95], an intelligent wireless battery-powered device that has been developed in order to improve beer taps efficiency through the data acquired and analyzed. The system, with respect to the previous cases, operates using a proprietary “I-Tap network” that continuously monitors the entire beer system through a web interface. It features advanced beer inventory and analytics behind consumption that, following their claim, allows for smarter business decisions across the board. In detail, besides basic parameters, it monitors also CO_2_ pressure, glycol temperatures, and more for real-time tracking and visualization on laptop or I-Tap smartphone app providing live information. Nevertheless, it allows inventory control reporting automatically which lines need new beers and can place kegs or CO_2_ cylinder orders autonomously by sending an email to the distributor. The device can auto shutdown the lines in the case of problems preventing waste, e.g., caused by involuntary emptying of beer lines, and represents a valuable tool for marketing by acquiring data regarding what brands were poured at what time and where. Finally, recent studies show a clear trend regarding the use of low-cost techniques using robotics, AI, computer vision, biometrics, and more also to assess the quality and consumer preferences regarding beverages [96,97].

### 4.4. IoB Technologies and Potential Use Summary

Of course, the available technologies are more than those cited by this work that, rather than find all the possible solutions available or under development, wants to reveal the possibilities and potential rising from the introduction of these in the beer panorama. Table 2 reports the potential impacts IoT systems might have on beer processing, logistics, and services. The table is structured following just three main categories (Intervention Level) since many technologies reported are potentially relevant for several sub-fields e.g., processing steps (see Figure 1).

## 5. Conclusions and Future Prospective

The IoB systems and the applications reviewed in the present work have the potential to bring benefits to the whole brewing chain from the beginning of the production process to the beer service. Benefits may arise range from energy and cost saving to increased management strategies including feedback coming from consumption analytics. It appeared as several breweries are already taking advantages from some of the presented technologies. These were firstly implemented by bigger brewing groups which have higher needs for product standardization and an advanced planning of activities. Often the technologies firstly implemented are proprietary, or developed following specific needs, and rely on cabled sensors and expensive infrastructures locally based. In this scenario the introduction of low-cost sensors, the presence of small elaboration boards, the increasingly popular IoT platforms offering both basic and advanced services at reasonable prices, are opening new possibilities for smaller size companies such as craft breweries. However, the potential benefits need to be carefully considered in relation to the implementation costs. A cost-related consideration regards the open-source nature of many devices and software. These normally have much lower price with respect to proprietary ones but do require increased management skills. In this regard we should distinguish between punctual implementations or complete systems. In the first case low technology skill may be enough while a whole optimized environment requires discrete work and testing. One strategy might be to implement a complete system step by step considering devices and ecosystems that follow the same standards or allow for high flexibility.

The first step is always to monitor and register parameters a second one regarding the creation of a decision support system (DSS) for practice optimization. A DSS for a craft brewery may represent a tool to optimize production and logistic since craft beers are generally more delicate and less stable with respect to industrial ones. For a big group, instead, a DSS (even AI based) might be useful to increase marketing strategies. Nowadays software are potentially powerful DSS tools that acquire data through sensors, share data with the supply chain and the retailers reaching even the consumers to retrieve feedback for each beer lot.

The trend that the future beer sector will face, will be a very fast increase in the use of IoB systems to drive efficiency, productivity, quality, and safety. This will be done by real-time monitoring and data-driven decision-making. These aspects that are and will become more and more crucial likewise in many other sectors, imply considerations regarding the data ownership and their standardization. The access price to IoB devices and apps is destined to significantly go down while their diversification on the market will grow. This will lead to a massive future implementation within all production levels, economically favoring the skilled companies that will implement correctly sized solutions in advance. Finally, many of the cited IoT apparatus, software, and hardware, can potentially be transferred to other sectors, near or far from the beer one, potentially producing the same benefits. If in the future, if standard protocols and data formats will be applied and embraced, different sectors could communicate and take advantages from data provided by other ones, e.g., production from logistics.

## Figures and Tables

**Table 1:** Scheme of the principal open IoT platforms and relative characteristics.

IoT Software Platform	Device Management	Integration	Security	Protocols for Data Collection	Analytics	Support for Visualizations	DB
Kaa IoT Platform	YES	Portable SDK available to integrate any particular platform, REST API	Link encryption (SSL), RSA key 2048 bits, AES key 256 bits	-MQTT; -CoAP; -XMPP; -TCP; -HTTP.	Real time IoT data analytics and visualization with: -Kaa; -Apache Cassandra; -Apache Zappelin.	YES	-MongoDB; -Cassandra; -Hadoop; -Oracle NoSQL.
SiteWhere	YES	REST API, Mule AnyPoint, and more	Link encryption (SSL), spring security	-MQTT; -AMQP; -STOMP; -Websockets; -Direct socket connections.	Real-time analytics (Apache Spark)	NO	-MongoDB; -HBase; -InfluxDB.
ThingSpeak	NO	REST MQTT APIs	Basic authentication	http	MATLAB analytics	NO	MySQL
DeviceHive	UNKNOWN	REST AP, MQTT APIs	Basic authentication using JSON Web Tokens (JWT)	-REST API; -WebSockets -MQTT.	Real-time analytics (Apache Spark)	YES	-PostgreSQL; -SAP Hana DB.
Zetta	NO	REST APIs	Basic authentication	http	Using Splunk	NO	Unknown
Distributed Services Architecture (DSA)	NO	REST APIs	Basic authentication	http	No	NO	ETSDB-embedded time series
Thingsboard.io	YES	REST APIs	Basic authentication	-MQTT; -CoAP; -HTTP.	Real time analytics (Apache Spark, Kafka)	NO	Cassandra
Thinger.io	YES	REST APIs	Link encryption (SSL/TLS) and basic authentication	-MQTT; -CoAP; -http.	Yes	NO	MongodB
WSo2	YES	REST APIs	Link encryption (SSL) and basic authentication	-HTTP; -WSO2 ESB; -MQTT.	Yes, WSO2 data analytics server	YES	-Oracle; -PostgreSQL; -MySQL or MS SQL.
Mainflux	YES	REST APIs	JWT encrypted and signed tokens, OAuth2.0, public key infrastructure (PKI) and client-side certificates	-HTTP; -MQTT; -WebSocket; -CoAP.	Yes (integrated) platform not confirmed	YES	-Cassandra; -MongoDB; -InfluxDB; -PostgreSQL.

**Table 2:** Examples of Internet of Beer (IoB) use cases and their potential impact within the three reported macro areas on intervention. The letters H and S indicate Hardware and Software, respectively. Hardware is normally supplied with its own software, sometimes open for developers to produce their own code.

Technology Intervention Level	Impact Benefits	Technologies	References
**Brewing process**	Energy use reduction; Product value increase; Workload reduction; Raw material monitoring and optimization; Problem identification; Increase of the production level; Time optimization; Product standardization; Improvement of temperature and fermentation control; Client management; Sales management; Real time parameters monitoring (pH, density, pressure, conductivity, dissolved oxygen and internal/external temperature; Automation of the fermentation process;	Cassia Networks Bluetooth Sensor; (H) Orchestratedbeer; (S) Beerifyme!; (S) Brewplanner; (S) Iconic^®^ BMS; (S) Vicinitybrew; (S) Brewtarget; (S) Beersmith 3; (S) Brewfather; (S) Promash; (S) Hobbybrew; (S) Plaato Airlock; (H, S) Gärspundmobil Speidel; (H, S) Ispindel Hydrometer; (H, S) Tilt™ Hydrometer; (H, S) Raspberry Pi board; (H) Arduino board; (H) Mybrewbot; (H, S) Smartpid; (H, S) Tzerobrew; (H, S) LORD Wireless Sensors; (H, S) Brewmonitor^®^ System; (H, S) Brewbot; (H, S) Grainfather; (H, S) Brewtools; (H, S) Speidel Braumeister (H, S) Sprowtlab; (H, S) Micro malting system BBC Inox (H, S) Arzaman S.R.L.; (H, S) Low-Cost Spectrophotometer; (H)	[28,29], [30,31], [32,33], [35,36], [37,38], [39,40], [41,42], [43,44], [45,46], [47,48], [49,50], [51,52], [53,54], [55,56], [57,58], [59,60], [61,62], [63,64], [65,66], [67,68], [16].
**Traceability and logistics**	Transport tracking; Logistic optimization Stock control and storage of raw materials; Monitoring the position, temperature, and pressure during transport; Sharing traceability data with the consumer and stakeholders; Protection against fraud and leakage of beer kegs; Transparency of supply chain; Reduce malfunctions along the beer supply chain;	Rfid,Nfc; (H) ROGUE Smart Sensors; (H) OVINTO Satellite Sensors; (H) Bock Chain and Qr Code Systems (H, S) Blockchain systems; (S) Big Data and AI; (S) Microsoft Azure Platform; (S)	[25,26], [69,70], [71,72], [73,74], [75,76], [25,26], [77,78], [79,80].
**Service, marketing, and consumption**	Consumer feedback; Customer consumption information; Reducing waste and fraud; Increase customer satisfaction; Optimize beer service; Real-time keg monitoring; Smarter business decisions.	Sensors applied to beer taps and bottle; (H, S) Taptronics (Pubinno); (H, S) Beerpoint Technology (based on Nfc System); (H, S) Robiotic (Rfid-enabled beer glass); (H, S) Beer button; (H, S) Kegtron (keg monitoring); (H, S) Ipourit (wristband with Rfid technology); (H, S) Wecheer.Io (intelligent bottle opener); (H, S) Functional thermochromic sensors and inks; (H, S) Simple Beer Service; (H, S) Kegbot (keg monitoring); (H, S) I-Tapr2 (tap and keg monitoring); (H, S)	[27,81] [82,83] [84,85] [86,87] [8,89][90,91] [92,93] [94,95].
